# Hospital Library

**Published:** 1919-11-08

**Authors:** H. M. Gaskell, C. Hagberg Wright

**Affiliations:** Hon. Secretarie, War Library. 48 Queen's Gardens, Lancaster Gate, London, W. 2.; Hon. Secretarie, War Library. 48 Queen's Gardens, Lancaster Gate, London, W. 2.


### THE EDITOR'S LETTER-BOX.

**[Correspondence on all subjects is invited, but we cannot in any way be responsible for the opinions expressed by our correspondents, who must give their name and address as a guarantee of good faith, but not necessarily for publication. Correspondents are reminded that brevity of style and conciseness of statement greatly facilitate early insertion.]**

### Hospital Library.

*To the Editor of* THE HOSPITAL.

SIR,—It has been decided by the Joint Committee of the Red Cross and Order of St. John to continue the work of the War Library for a term of five years, under the name of "The British Red Cross and Order of St. John War and Peace Hospital Library," at 48 Queen's Gardens, Lancaster Gate, London, W. 2. This decision is in response to an appeal signed by those who had the widest experience of the great and beneficial work done for the sick and wounded by the War Library during the war. Literature in hospitals is always needed, in war and in peace. Large numbers of men require treatment for an indefinite period. In sending you our grateful thanks for your gifts of the past, we ask you to continue to send us your spare books, magazines, and illustrated papers to the above address, from which centre they will be distributed to the hospitals and hospital-ships in Great Britain, and our Armies of Occupation. Our funds do not permit us to purchase largely, and it is hard to say "No" when suffering men write for books and magazines. We must in the future depend largely on the public remembering us and our needs; therefore please constantly send us a book or magazine when it is no longer needed.—Yours truly,

H. M. GASKELL,
C. HAGBERG WRIGHT,
Hon. Secretaries, War Library.

48 Queen's Gardens, Lancaster Gate, London, W. 2.

[We hope that our readers will, and thus incidentally improve the miserable shelf which usually is misnamed "Our Hospital Library."—ED. "THE HOSPITAL."]

